# Ultra-high field MRI of human hippocampi: Morphological and multiparametric differentiation of hippocampal sclerosis subtypes

**DOI:** 10.1371/journal.pone.0196008

**Published:** 2018-04-18

**Authors:** Clarissa Gillmann, Roland Coras, Karl Rössler, Arnd Doerfler, Michael Uder, Ingmar Blümcke, Tobias Bäuerle

**Affiliations:** 1 Institute of Radiology, University Hospital Erlangen, Erlangen, Germany; 2 Institute of Neuropathology, University Hospital Erlangen, Erlangen, Germany; 3 Department of Neurosurgery, University Hospital Erlangen, Erlangen, Germany; 4 Department of Neuroradiology, University Hospital Erlangen, Erlangen, Germany; University of Modena and Reggio Emilia, ITALY

## Abstract

The aim of the present study is to differentiate subtypes of hippocampal sclerosis (HS) using ex vivo ultra-high field magnetic resonance imaging (MRI). Included were 14 surgically resected hippocampi of patients with medically intractable temporal lobe epilepsy. The resected hippocampi were histologically categorized into subtypes of hippocampal sclerosis (HS type 1 (n = 10), HS type 2 (n = 2) and no-HS (n = 2)) and subsequently scanned on a preclinical 7T MRI acquiring T2-weighted morphology, relaxometry and diffusion tensor imaging. On the morphological images, the pyramidal cell layer (PCL) of the hippocampus was segmented and the following parameters were derived: T2 signal intensity, T1-, T2- and T2*-relaxation times, apparent diffusion coefficient (ADC), fractional anisotropy (FA) and mean diffusivity (MD). Furthermore, the area of the PCL was determined, as well as the parameter product which refers to the widths of the PCL parallel and perpendicular to the stratum moleculare. Spearman correlation coefficient was used to demonstrate relationships between MR-parameters and type of sclerosis. In comparison to no-HS specimens, the PCL was significantly narrower in HS type 1 and HS type 2 hippocampi. This narrowing affected the entire cornu ammonis sector (CA) 1 in HS type 1, while it was limited to the upper half of CA1 in direction to CA2 in HS type 2. The parameter product median increased from 0.43 to 1.67 and 2.91 mm^2^ for HS type 1, HS type 2 and no-HS, respectively. Correlation coefficients were significant for the PCL parameters product (0.73), area (0.71), T2*-time (-0.67), FA (0.65) and ADC (0.55). Our initial results suggest that HS type 1, HS type 2 and no-HS subtypes can be distinguished from each other using ex vivo UHF MRI based on T2-weighted morphologic images and the assessment of the parameter product. Upon clinical translation, UHF-MRI may provide a promising technique for the preoperative differentiation of HS subtypes in patients.

## Introduction

Hippocampal sclerosis (HS) is observed in up to 70% of patients with mesial temporal lobe epilepsy (TLE) [1]. Up to one third of TLE patients suffer from intractable or drug-resistant epilepsy, and based on the evidence of hippocampal involvement in seizure generation, surgical resection of the ipsilateral hippocampus is the standard of care for these patients [2–4].

According to the International League against Epilepsia (ILAE), HS can be histologically divided into four subtypes based on specific patterns of neuronal cell loss in the pyramidal cell layer (PCL) [5]. Different HS subtypes may have different prognosis for postoperative seizure and memory outcome [6–11]. A differentiation of HS subtypes in preoperative imaging may therefore lead to a more accurate TLE diagnosis, improved treatment selection and better patient management [12].

Magnetic resonance imaging (MRI) is crucial for diagnosing hippocampal sclerosis in TLE patients. Typical features of HS are reduced hippocampal volume, increased signal intensity of the PCL on T2-weighted and T2-fluid attenuation inversion recovery (FLAIR) images and loss of internal architecture [13,14]. Furthermore, an increase of the mean diffusivity (MD) and a reduction of fractional anisotropy (FA) have been reported in patients with HS [15–17].

While current clinical protocol focuses on differentiating HS from no-HS, little is known about the MR characteristics of different HS subtypes. One reason for this is the limited ability of clinical 1.5T and 3.0T MRI scanners to image the internal architecture of the hippocampus due to spatial resolution and tissue contrast [12–14]. As a result, UHF MRI at 7.0 Tesla may be a promising technique to non-invasively and preoperatively assess HS characteristics in patients.

To evaluate the potential of UHF MRI, we chose an ex vivo UHF MRI setting by making use of strong magnetic fields, fast and powerful gradients, long scan times and the absence of motion, thus allowing for micrometer-scale resolutions, strong tissue contrasts and an increased signal-to-noise ratio in multiparametric studies. The present study therefore aims to address the morphology, relaxometry and diffusion metrics of surgically resected HS type 1, HS type 2 and no-HS hippocampi. The question we focus on in this ex vivo study is whether HS characteristics can be visualized and quantified by morphological and multiparametric UHF MRI.

## Materials and methods

### Hippocampal specimens

This study includes 14 patients diagnosed with medically intractable TLE who underwent surgery for the relief of seizures at University Hospital Erlangen after imaging and electroclinical characteristics had shown evidence of hippocampal involvement in seizure generation.

Directly after surgery, resected hippocampi were fixed in a 4% paraformaldehyde buffered solution, washed with phosphate buffered saline (PBS) and submitted for neuropathological examination. The specimens were diagnosed with HS type 1 (n = 10), HS type 2 (n = 2) and no-HS (n = 2) according to ILAE guidelines. All procedures were approved by the local ethics committee (Ethik-Kommission der Friedrich-Alexander-Universität Erlangen-Nürnberg, approval number: 92_14B). Participant’s written informed consent was obtained.

### MRI

For imaging, hippocampal specimens were fixed in a standardized procedure for 24 h in paraformaldehyde and afterwards embedded in 1.5% agarose. Imaging war performed on a preclinical 7T MRI (ClinScan 70/30, Bruker, Ettlingen, Germany, gradient amplitude: 290 mT/m, slew rate: 1160 T/m/s) using a volume resonator radiofrequency coil (Bruker, Ettlingen, Germany). For technical reasons, three samples (n = 1 respectively of HS type 1, HS type 2 and no-HS) were imaged with a surface coil. The imaging protocol included T2-weighted morphology, relaxometry and diffusion tensor imaging (DTI). The field of view (30x30 mm^2^), slice thickness (300 μm) and image center were identical for all sequences. Morphological images were acquired using a 3D T2-weighted turbo spin echo sequence (turbo factor: 7, number of phase encoding steps: 707, repetition time (TR)/echo time (TE): 8520/95 ms, matrix: 704x704, averages (av): 4, acquisition time (TA): 207 min per slice). Relaxometry was acquired using a 3D fast low angle shot-sequence (TR/TE: 50/2.5, flip angles: 8° and 42°) for T1-times. A 2D spin-echo sequence (TR: 7210 ms, TEs: 10.3–51.5 ms in 5 intervals) was used for T2-times and a 2D-gradient echo sequence (TR: 100 ms, TEs: 4–40 ms in 10 intervals) for T2*-times, each with 3 averages, a matrix of 576x576 and acquisition times of 5, 210 and 14 minutes per slice, respectively. Diffusion tensor imaging (DTI) was performed with six b-values (b = 0, 200, 400, 600, 800, 1000 s/mm^2^) in 265 directions using a 2D echo planar imaging sequence (TR/TE: 8000/50 ms, av: 3, matrix: 100x100, TA: 8.5 h, per slice).

During post-processing, voxel-based three-dimensional parameter maps of T1-, T2- and T2*-times, ADC, MD and FA were calculated for each hippocampus (SyngoVia software, Siemens, Erlangen, Germany).

### Image analysis

Image analysis was performed using an Osirix Dicom Viewer (aycan Osirix, USA) in conjunction with Chimaera's segmentation tool (Chimaera GmbH, Erlangen, Germany). The pyramidal cell layer (PCL) was segmented on the first slice of the T2-weighted morphological image stack on which the hippocampus was homogeneously visible. The manually drawn segmentation covered the four cornu ammonis sectors (CA1, CA2, CA3 and CA4) of the PCL. The CA1/subiculum border was determined according to Mueller et al [18] by drawing a line perpendicular to the edge of the subiculum touching the medial border of the hippocampus. The segmentation mask was transferred to the respective parameter maps and the mean values of T2 signal intensity (T2Si), T1-, T2- and T2*-times, ADC, MD and FA averaged over the segmented area of the PCL were calculated. As additional morphological parameters, the area of the PCL was extracted and the parameter product was quantified by measuring two widths of the PCL: The first width, "a", was defined as the width of the PCL in extension to stratum moleculare and "b" was measured as the width of the PCL perpendicular to stratum moleculare. The parameter product was then calculated as a*b.

### Statistical analysis

Statistical analysis was performed using statistic software R [19]. Spearman rank correlation coefficients with a type of sclerosis as an independent variable were calculated for each MR-parameter, numerically coding the different subtypes as 1, 2 and 3 (HS type 1, HS type 2 and no-HS, respectively). Differences in the parametric quantities between HS subtypes were tested by a paired, 2-sided Wilcoxon test and p<0.05 was considered statistically significant.

## Results

The study results can be divided into a qualitative analysis, based on morphologic T2-weighted MR images, and a quantitative part, including the parameters area (mm^2^), product (mm^2^), T2 signal intensity (a.u.), T1-time (ms), T2-time (ms), T2*-time (ms), FA (a.u.), ADC (*10^−6^ mm^2^/s) and MD (*10^−6^ mm^2^/s).

### Qualitative analysis

**Fig 1:**
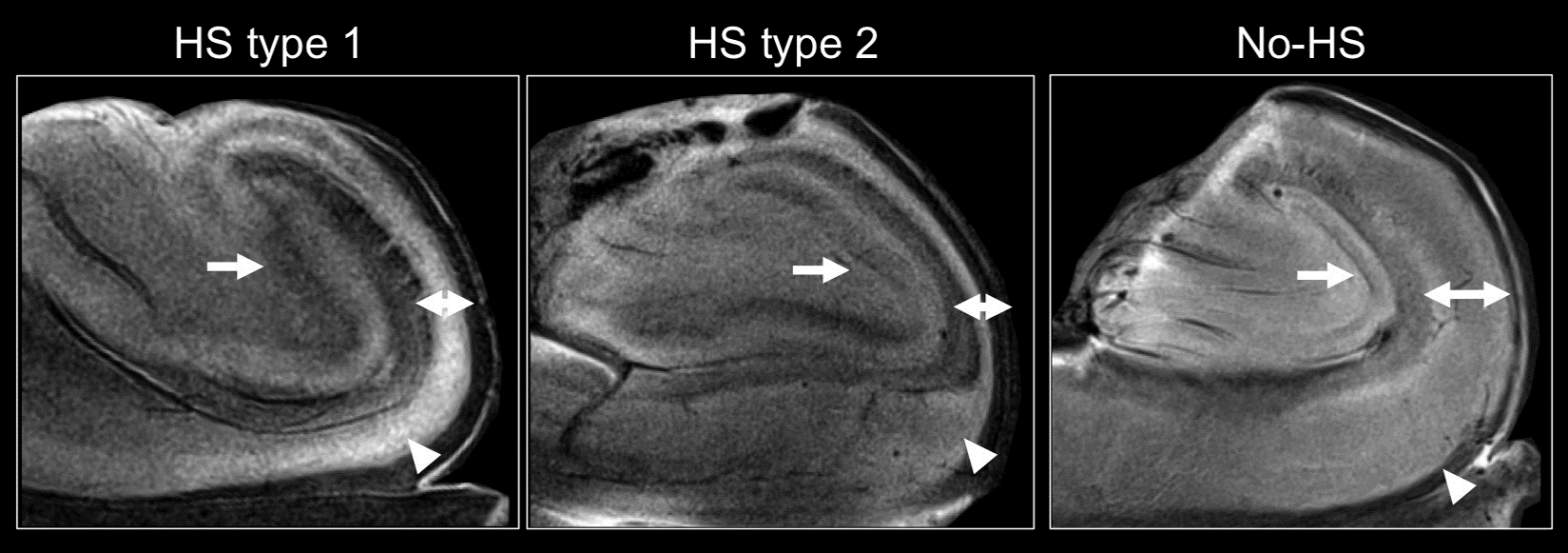
T2-weighted morphological MR-images of representative HS type 1, HS type 2 and no-HS hippocampi. Image resolutions are 43x43x300 μm, respectively. The PCL is significantly narrower in HS type 1 and HS type 2 in comparison to no-HS hippocampi (two-sided arrows). With respect to CA1, the narrowing affects the whole sector in HS type 1 specimens, while it is limited to the upper half of CA1 in direction to CA2 in HS type 2 (triangles). The dentate gyrus (DG) is broadened and focally not discernible in HS type 1 and HS type 2, whereas it can be identified as a distinct thin black line (arrow) in no-HS specimens.

Fig 1 depicts T2-weighted morphologic MR images of representative HS type 1, HS type 2 and no-HS specimens. The image resolutions are 43x43x300 μm. In HS type 1 and HS type 2, the PCL is significantly narrower as compared to no-HS, gliosis-only hippocampi. The narrowing affects the whole sector cornu ammonis (CA) 1 in HS type 1 hippocampi, while in HS type 2, only the upper half of CA1 in direction to CA2 is atrophic. No-HS specimens do not show any signs of atrophy. Narrowed/atrophic areas of the PCL appear hyper-intense on T2-weighted morphologic images. In sclerotic specimens (HS type 1 and HS type 2), the dentate gyrus (DG) is broadened and focally indiscernible and has an ill-defined boundary with the molecular layer. In no-HS hippocampi, DG can be identified as a distinct thin black line.

### Quantitative analysis

**Fig 2:**
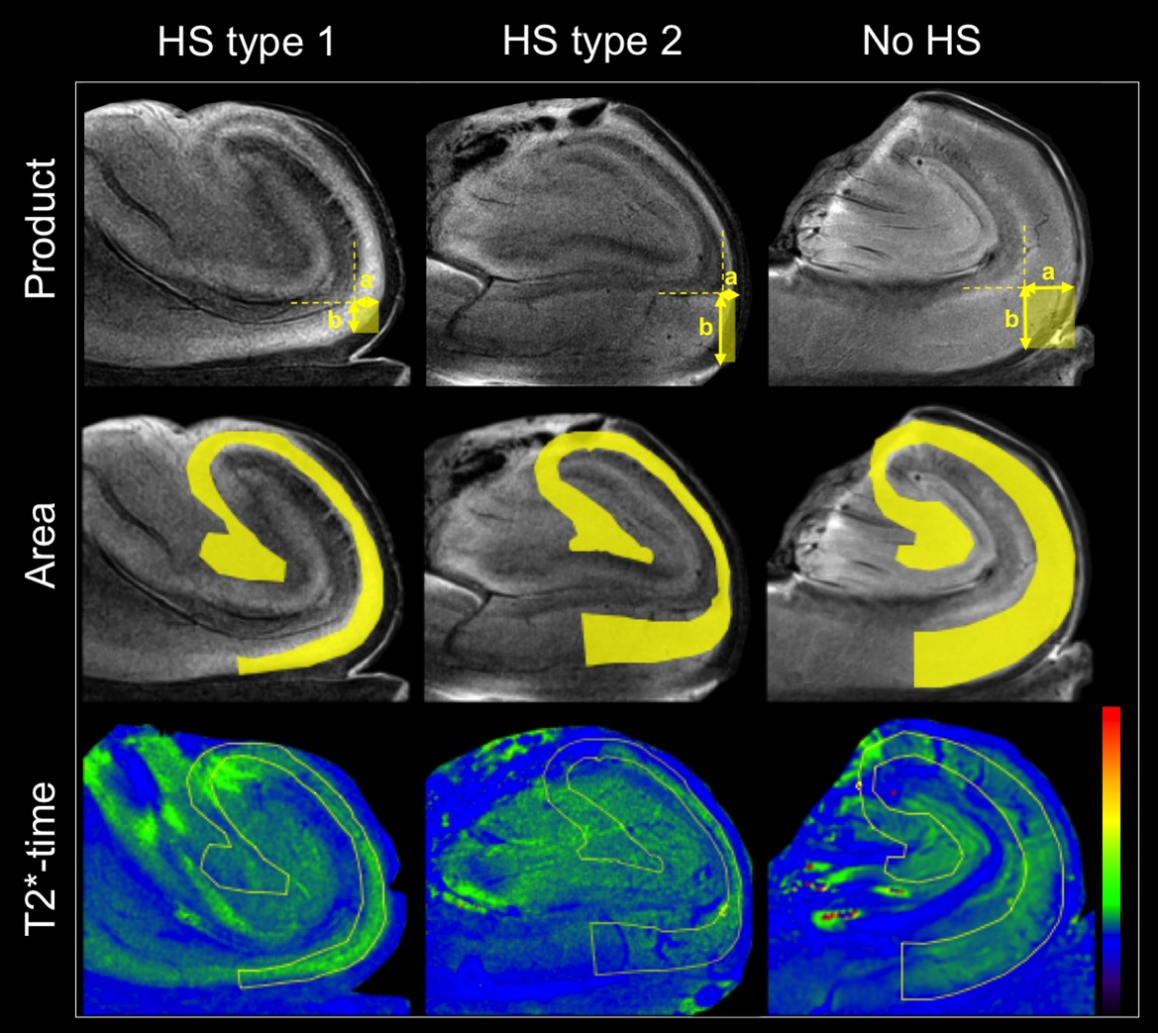
Illustration of the quantitative MR-parameters product and area, including color-coded maps of T2*-time of HS type 1, HS type 2 and no-HS hippocampi. The parameter product, as is exemplarily shown in the upper row, is derived by multiplying widths a and b of the PCL and can be illustrated as a rectangle. The parameter area (second row) refers to the area of the PCL, which is skirted by the segmentation mask, shown in yellow. T2*-time is determined as mean value within the segmentation mask, defining the area of the PCL.

**Fig 3:**
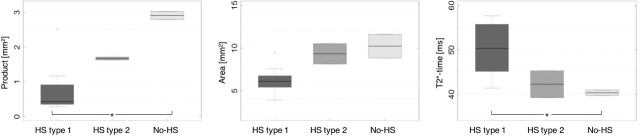
Boxplots of the parameters product, area and T2*-time for HS type 1, HS type 2 and no-HS hippocampi.

Quantitative parameters were determined for each hippocampus when segmenting the pyramidal cell layer. The following results were obtained by grouping the specimens according to their neuropathologic diagnosis: The median value of the parameter product increases from 0.43 to 1.67 and 2.91 mm^2^ for HS type 1, HS type 2 and no-HS, respectively. Here, significant differences were found between HS type 1 and no-HS. The parameter area increases from HS type 1 to HS type 2 and no-HS. The relaxation time T2* decreases from 50.3 (HS type 1) to 42.3 (HS type 2) to 40.3 ms (no-HS), with significant differences between HS type 1 and no-HS. FA increased for HS type 1, HS type 2 and no-HS, while ADC and MD show decreasing tendencies. Spearman Rank correlation coefficients demonstrated significant relationship between type of sclerosis and the MR-parameters product (0.73), area (0.71), T2* (-0.67), FA (0.56) and ADC (-0.55). All other parameters including T2Si (-0.45), T1-time (0.15), T2-time (0.15) and MD (-0.46) did not reach significance. The results of all quantitative parameters assessed in this study are summarized in Table 1. Metadata are available as Supporting information (S1 Table). MR-images of product, area and T2*-time are shown in Fig 2. Boxplots of the quantitative analysis are depicted in Fig 3.

**Table 1:** Quantitative analysis of MR-parameters.

	HS type 1	HS type 2	No-HS	R
**Area [mm**^**2**^**]**	6.1 (5.4/6.6)	9.3 (8.1/10.5)	10.2 (8.8/11.6)	**0.71**
**Product [mm**^**2**^**]**	0.43 (0.35/0.91)	1.67 (1.63/1.71)	2.91(2.79/3.03)	**0.73**
**T2Si [a.u.]**	359.6 (306.8/420.5)	221.0 (154.8/287.3)	239.7 (153.0/326.4)	-0.45
**T1-time [ms]**	972.6 (639.3/1051.5)	758.9 (450.8/1067.1)	1183.9 (870.1/1497.7)	0.15
**T2-time [ms]**	99.0 (93.9/102.4)	123.6 (82.4/164.8)	127.4 (69.7/185.1)	0.15
T2*-time [ms]	50.3 (45.1/55.8)	42.3 (39.2/45.3)	40.3 (39.7/41.0)	**-0.67**
**FA [a.u.]**	0.145 (0.119/0.178)	0.290 (0.185/0.396)	0.207 (0.165/0.248)	**0.56**
ADC [*10^**−6**^ **mm**^**2**^**/s]**	569.6 (535.5/755.2)	457.6 (413.5/501.7)	480.1 (388.0/572.2)	**-0.55**
MD [*10^**−6**^ **mm**^**2**^**/s]**	188.5 (149.8/209.6)	132.1 (114.3/150.0)	131.2 (91.0/171.4)	-0.46

Quantitative analysis of MR-parameters given in median values with first (25%) and third (75%) quartiles. Spearman Rank correlation coefficients R of the parameters with type of sclerosis are shown in the last column with significant correlations printed in bold.

## Discussion

In patients suffering from medically intractable TLE, the surgical outcome and prognosis for post-operative seizure freedom are associated with different subtypes of hippocampal sclerosis that can currently only be diagnosed and categorized by histological analysis. As a first step towards a preoperative subtype differentiation using non-invasive imaging methods, we investigated the morphological and quantitative characteristics of surgically resected HS type 1, HS type 2 and no-HS hippocampi by multiparametric ex vivo UHF MRI.

Our results suggest that HS type 1, HS type 2 and no-HS gliosis only hippocampi can be distinguished from each other based on specific morphological patterns of the PCL, which we were able to depict in high-resolution T2-weighted MR-images acquired with long repetition times and the resulting strong tissue contrasts. These morphological differences can be quantitatively captured by the parameter product, which we have introduced and defined in the present study. Furthermore, the morphological parameters product and area, T2*-time, FA and ADC yielded significant correlation coefficients depending on the type of sclerosis.

The median values of T2*-time in the PCL increased from HS type 1 to HS type 2 and no-HS hippocampi. This tendency was also observed in a recent in vivo study by Santyr et al 20[], who found R2* (= 1/T2*) to be significantly lower in the entire hippocampus of HS patients when compared to controls. However, the authors did not find a significant decrease of R2* for patients with HS in comparison to patients diagnosed with no-HS [20].

As T2* relaxation results from susceptibilities among tissues, this increase could correlate with sclerosis-induced tissue homogenization in the PCL, similarly to an increase in the T2 signal and T2 relaxation time. It has been formerly suggested that the later reflect gliosis [21–23] and are commonly associated with astrogliosis [15,18,24].

Our finding of FA values is clearly lower in the PCL of HS type 1 when compared with HS type 2 and no-HS hippocampi, which may reflect stronger neuronal cell loss. This result concurs with clinical studies that found FA to be decreased in patients with HS in comparison to patients without HS [15–17]. However, a recent study by Coras et al, investigating HS subtypes by ex vivo MRI, claimed that FA values are uninformative [25]. This discrepancy with our finding might be explained by the fact that the DTI sequence of Coras et al was based solely on one b-value (1200 s/mm^2^) and that only126 diffusion directions were investigated, whereas the DTI sequence used in this study included six b-values and 256 diffusion directions.

In terms of a histological interpretation, an increase of ADC-values in stronger pathologies may reflect a loss of neurons, respectively the shrinkage of neuronal bodies, which may lead to less restricted water diffusion and therefore higher diffusivity [10].

T2 signal intensity, as well as T1 and T2 relaxation times, did not show significant correlation with the type of sclerosis investigated in our study. T2 relaxation time in particular has been shown to be increased in patients with TLE, in vivo as well as ex vivo [26]. For this reason and since the parameter is a quantitative measure for T2-hyperintensity, we would have expected T2 time to be of higher significance. However, the small voxel size of our imaging protocol probably hampered a reliable calculation of T2-time since the signal was obtained from very few spins. We expect this parameter to gain more impact the closer we get to clinical resolutions as the spin echo in one voxel will then be obtained from a bigger group of spins precessing with different frequencies and thus showing a stronger transverse decay.

Our study has several limitations: The sample size of our specimens, especially for HS type 2 and no-HS hippocampi is very small. A higher number of HS type 2 and no-HS specimens or even HS type 3 hippocampi would increase the power of our study, however, as these are very rare forms of HS, no further specimens were available at our institution. Therefore, our results must be interpreted as preliminary and need to be confirmed in a larger patient collective. Furthermore, according to ILAE guidelines, the classification into different HS subtypes is based on distinct scores for neuronal cell loss in the individual subfields of the PCL (CA1-CA4 and the dentate gyrus). It therefore remains to be elucidated as to how far our observation that different HS subtypes express different atrophy patterns in the PCL can be correlated to histological data.

When performing ex vivo MRI, the impact of fixation on MR-parameters has to be considered. Formalin fixation has been reported to cause a strong decrease of all relaxation times [27–31]. In a recent study, Santos et al reported a decrease of T2-time in the hippocampi of TLE patients to the order of 20% after 7 days of formalin fixation [26]. Fixation also causes a loss of water, influencing diffusion metrics [10]. To study this effect, Goubran et al compared in vivo versus ex vivo FA and MD measurements in hippocampal specimens [12]. They concluded that absolute values were decreased for ex vivo, the relative data, however, was concordant for in vivo and ex vivo measurements.

Our data may provide a reference for the adaptation of in vivo imaging protocols on ultra-high field scanners. A first in vivo feasibility study of 7T MRI of hippocampal sclerosis by Stefanits et al [32] demonstrated the potential of UHF-MRI for the detection of pathology in the CA subfields with sensitivity and specificity values of up to 100%.

## Conclusion

Our initial results suggest that HS type 1, HS type 2 and no-HS subtypes can be qualitatively and quantitatively distinguished from each other using ex vivo UHF MRI based on T2-weighted morphological images and the assessment of the parameter product. T2*-relaxation time and diffusion metrics may additionally contribute to a differentiation of HS subtypes.

In terms of a potential clinical translation, UHF MRI may be a promising technique to determine HS subtypes preoperatively in patients.

## Supporting information

S1 Table (PDF)
