# Supplementary material for: Ultra-high field MRI of human hippocampi: Morphological and multiparametric differentiation of hippocampal sclerosis subtypes
Source: PLoS One. 2018 Apr 18;13(4):e0196008. doi: 10.1371/journal.pone.0196008 (PMC5906020; doi:10.1371/journal.pone.0196008)
Supplement: S1 Table — (PDF) [file pone.0196008.s001.pdf]

Sample	HS Type	Product [mm^2]	Area [mm^2]	T2Si [a.u.]	T1-time [ms]	T2-time [ms]
HC001	I	0.41	6.02	123.71	331.29	53.67
HC002	no-HS	2.79	8.84	152.95	1497.74	185.13
HC003	II	1.71	10.54	154.78	1067.05	164.82
HC005	I	0.35	4.79	306.79	639.35	58.26
HC006	I	0.46	7.59	420.48	801.10	131.11
HC007	no-HS	3.03	11.64	326.35	870.09	69.66
HC008	I	0.31	5.42	384.16	983.30	101.75
HC009	I	0.91	6.76	324.60	485.16	135.83
HC010	I	0.35	6.62	366.11	961.97	93.90
HC011	I	1.16	6.19	445.42	1051.51	98.40
HC012	II	1.63	8.14	287.32	450.80	82.35
HC013	I	2.51	9.47	439.10	1112.56	96.16
HC014	I	0.52	5.77	353.02	996.66	99.67

T2*-time [ms]	FA [a.u.]	ADC [*10^{-6} mm^2/s]	MD [*10^{-6} mm^2/s]
51.28	0.14	755.23	181.03
40.97	0.25	387.99	273.11
39.22	0.40	413.52	342.92
41.38	0.20	535.55	523.22
56.91	0.12	566.31	621.60
39.67	0.17	572.24	514.14
42.61	0.19	572.81	449.30
49.33	0.10	629.14	579.89
45.13	0.14	665.80	551.15
55.77	0.12	527.89	705.68
45.30	0.19	501.71	449.96
45.74	0.15	506.86	628.74
57.64	0.18	576.39	659.65
